# Prevalence of trachoma in Somali region, Ethiopia: results from trachoma impact surveys in 50 woredas

**DOI:** 10.1093/inthealth/ihad063

**Published:** 2023-12-04

**Authors:** Getachew Gebreselassie, Kasahun Negash, Sentayehu Tsegaye, Misrak Makonnen, Baye Deneke, Muluken Desalegn, Emma M Harding-Esch, Anna Harte, Anthony W Solomon, Sarah Boyd, Ana Bakhtiari, Mussie Abdosh Hassen, Abdulahi Hambali, Michael Dejene, Colin Beckwith, Fentahun Tadesse, Fikre Seifu, Genet Kiflu, Fikreab Kebede

**Affiliations:** Amref Health Africa, Addis Ababa, Ethiopia; Amref Health Africa, Addis Ababa, Ethiopia; Amref Health Africa, Addis Ababa, Ethiopia; Amref Health Africa, Addis Ababa, Ethiopia; Amref Health Africa, Addis Ababa, Ethiopia; Amref Health Africa, Addis Ababa, Ethiopia; Clinical Research Department, London School of Hygiene & Tropical Medicine, London, United Kingdom; Clinical Research Department, London School of Hygiene & Tropical Medicine, London, United Kingdom; Global Neglected Tropical Diseases Programme, World Health Organization, Geneva, Switzerland; Task Force for Global Health, Decatur GA, USA; Task Force for Global Health, Decatur GA, USA; Somali Regional Health Bureau, Jijiga, Ethiopia; Somali Regional Health Bureau, Jijiga, Ethiopia; Sightsavers, Haywards Heath, United Kingdom; Sightsavers, Haywards Heath, United Kingdom; Sightsavers, Haywards Heath, United Kingdom; Ethiopia Ministry of Health, Addis Ababa, Ethiopia; Ethiopia Ministry of Health, Addis Ababa, Ethiopia; Ethiopia Ministry of Health, Addis Ababa, Ethiopia

**Keywords:** Ethiopia, prevalence, Somali region, trachoma, trichiasis, tropical data

## Abstract

**Background:**

Following interventions to eliminate trachoma in Somali region, Ethiopia, we aimed to re-estimate the prevalence of trachomatous trichiasis (TT) and trachomatous inflammation—follicular (TF) at woreda level and identify the factors associated with the disease.

**Methods:**

We implemented cross-sectional community-based surveys in 50 trachoma-endemic woredas, using a standardized survey. Households were the secondary sampling unit. Surveys were undertaken through a combination of interviews of household heads and direct inspection of water, sanitation and hygiene (WASH) access, plus clinical evaluation of eligible household members for TT and TF.

**Results:**

Overall, 41 (82%) of the 50 woredas had met the WHO-recommended active trachoma elimination threshold (prevalence of TF <5% in 1–9-y-olds) and 42 (84%) had met the TT threshold (prevalence of TT unknown to the health system <0.2% in ≥15-y-olds). Only 18% of households had access to an improved drinking water source within a 30-min trip and only 25% had an improved latrine.

**Conclusions:**

Additional rounds of antibiotic mass drug administration, plus interventions to enhance facial cleanliness and improve the environment, are required in nine woredas. TT surgical campaigns are needed in eight woredas. Greater access to WASH is required across all the woredas that were surveyed.

## Introduction

Ocular infection with particular strains of the bacterium *Chlamydia trachomatis* causes trachoma. It is a disease characterized by inflammation of the conjunctiva, most commonly in children, that can lead to scarring, opacity of the cornea and blindness in later life.^1^ Trachoma is largely found in poor, rural communities in low-income countries, where access to water, sanitation and healthcare is inadequate.^2^

In 1996, WHO, in collaboration with national health ministries academics, donors and non-governmental organizations (NGOs), founded an Alliance to eliminate trachoma as a public health problem (Global Elimination of Trachoma by 2020).^3^ The strategy to achieve this goal is known as SAFE, and comprises four types of measures: **S**urgery for trichiasis (to reposition eyelashes that touch the eyeball, potentially abrading the cornea), **A**ntibiotics to clear infection, and **F**acial cleanliness and **E**nvironmental improvement (particularly fly control and improved access to water) to reduce *C. trachomatis* transmission.^3,4^

To determine whether or not public health-level interventions are needed against trachoma, WHO recommends prevalence surveys to be conducted at evaluation unit (EU) level. In general, an EU is a district (known as a woreda in Ethiopia), which for trachoma elimination purposes WHO defines as ‘the normal administrative unit for health care management, consisting of a population unit between 100 000–250 000 persons’.^5^ Globally, in June 2022, 1649 EUs had a prevalence of trachomatous trichiasis (TT) in ≥15-y-olds above the corresponding elimination prevalence threshold of 0.2%,^2^ and a total population of 125 million qualified for implementation of the A, F and E components of the SAFE strategy. (The latter figure is based on the combined population of districts having a prevalence of the active trachoma sign ‘trachomatous inflammation—follicular’ [TF] in 1–9-y-olds above the elimination prevalence threshold of 5%.^2^) A majority (84%) of the 125 million people were in WHO's African Region, with 52% of the global total being in Ethiopia.

Somali region is one of the 11 regional states that make up the Federal Democratic Republic of Ethiopia. The region is divided into 11 zones, 93 woredas and six city administrations. In 2007, Somali region was estimated to have a total population of 4 445 219, consisting of 2 472 490 men and 1 972 729 women; urban inhabitants represented 14% of the population, and a further 38% were pastoralists. In addition, eight refugee camps and a transit center accommodate a collective 212 967 refugees from Somalia.

From 2013 to 2017, standardized baseline trachoma prevalence surveys took place in the Somali region of Ethiopia with Global Trachoma Mapping Project (GTMP) (pre-2017) and Tropical Data (2017) support, with the aim of identifying districts that needed interventions.^6–8^ The prevalence of TF ranged from 0.97% to 38.1%. The 27 woredas with TF prevalence from 5.0% to 9.9% received one round of antibiotic (either oral azithromycin [Zithromax] or topical tetracycline eye ointment) mass drug administration (MDA) during December 2019 and January 2020, and the 23 woredas with TF prevalence from 10% to 29.9% received three rounds of antibiotic MDA from December 2019 to December 2021. The mean reported all-age population coverage was 88%. In addition, Amref Health Africa, in collaboration with the Ministry of Health and Regional Health Bureau, implemented a project (2020–2023) in all trachoma-endemic woredas of Somali region to address the F and E components of the SAFE strategy.

The aim of the current study was to re-estimate trachoma prevalence in the 50 woredas of Somali region that received the WHO-recommended number of MDA rounds, to determine whether interventions needed to continue or could be stopped. The specific objectives were to assess the prevalence of TF in 1–9-y-olds and TT in ≥15-y-olds at woreda level and to obtain population-based data on access to water, sanitation and hygiene (WASH).

## Materials and Methods

Surveys were conducted with Tropical Data support, following WHO-recommended methodologies and building on the methods and technologies developed as part of the GTMP.^9^

### Training of graders and recorders

Tropical Data's standardized 5-d basic and refresher training sessions were conducted immediately before each series of surveys.^10^ Grader training was focused on eye examination for signs of trachoma using the WHO simplified trachoma grading system.^11^ Grader trainees who passed a slide-based inter-grader agreement (IGA) test with a kappa score of ≥0.7 took a field-based IGA test, in which they graded 50 eyes of children and again needed a kappa of ≥0.7 for TF grading compared with a Tropical Data-certified grader trainer. Graders were also trained to look for the presence or absence of trichiasis, trachomatous scarring (TS) and trachomatous inflammation—intense (TI), but were not tested on diagnostic accuracy of these signs. Likewise, recorder trainees underwent intensive training on survey processes, recognition of WASH infrastructure and data recording using the Tropical Data Android phone app. They were eligible for survey deployment if they achieved ≥90% in the recorder reliability test.

Thirty-nine new trainees participated in the basic training program before starting survey work. Nineteen graders and 20 recorders who had been previously trained but had not attended basic or refresher training within the preceding 12 mo attended refresher training before starting fieldwork. Teams were supervised by field supervisors; one field supervisor was responsible for the supervision of 6–7 teams.

### Survey design

Surveys employed a community-based cross-sectional design with a combination of interviews of heads of households to discuss access to WASH, direct inspect of WASH facilities and clinical examination of eligible members of the household for trichiasis (upper and lower eyelid separately), TS (for individuals with trichiasis, upper or lower eyelid), TF and TI. A separate survey was undertaken for each woreda.

### Sample size determination and sampling

The sample size for each survey was determined using the single population proportion for precision formula. The design effect was set at 2.63^11^ and an inflation factor of 1.2 was applied to account for non-response. To measure a prevalence of TF of 4% with a precision of ±2% at the 95% confidence level, a total of 1164 children aged 1–9 y would be needed.^9^ For woredas with a population size of <100 000, the sample size was corrected for a small, finite population.

The number of clusters needed from each EU was determined by dividing the total targeted number of children aged 1–9 y by the product of the average number of households a team could comfortably visit per day (30 households) and the estimated mean number of 1–9-y-olds per household (1.5).^13^ Thus, for woredas with population sizes of ≥100 000, 26 clusters were needed per survey. For woredas with population sizes of <100 000, the sample size was corrected for a finite population.

A two-stage cluster sampling technique was used. In the first stage, 26 (or fewer for smaller woredas) kebeles (the smallest administrative division) were selected using a probability proportional to population size approach. However, for the 23 EUs surveyed in 2021–2022, 30 clusters were selected on the basis that statistical modeling has shown that surveying 30 clusters provides acceptable precision around the TT prevalence estimate.^14^ In the second stage, compact segment sampling was employed to pick 30 households from the selected kebele. All individuals aged ≥1 y and living in sampled households were invited to be involved.

**Figure 1.**
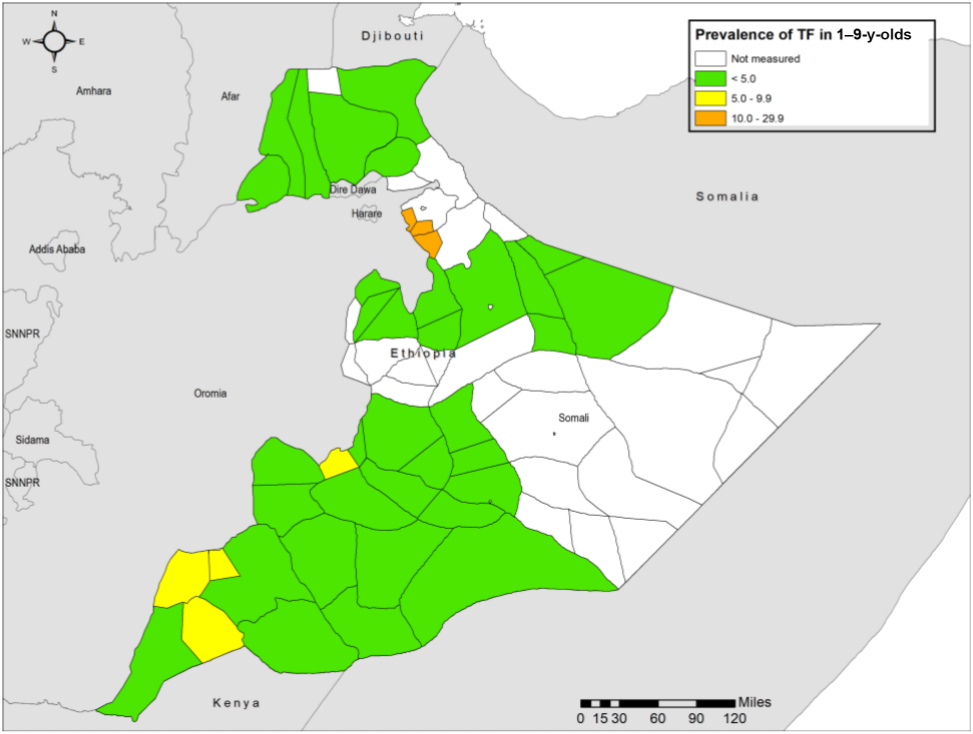
Prevalence of trachomatous inflammation—follicular (TF) in 1–9-y-olds, in trachoma impact surveys, Somali region, Ethiopia, 2020–2022. The boundaries and names shown and the designations used on this map do not imply the expression of any opinion whatsoever on the part of the authors, or the institutions with which they are affiliated, concerning the legal status of any country, territory, city or area or of its authorities, or concerning the delimitation of its frontiers or boundaries.

**Figure 2.**
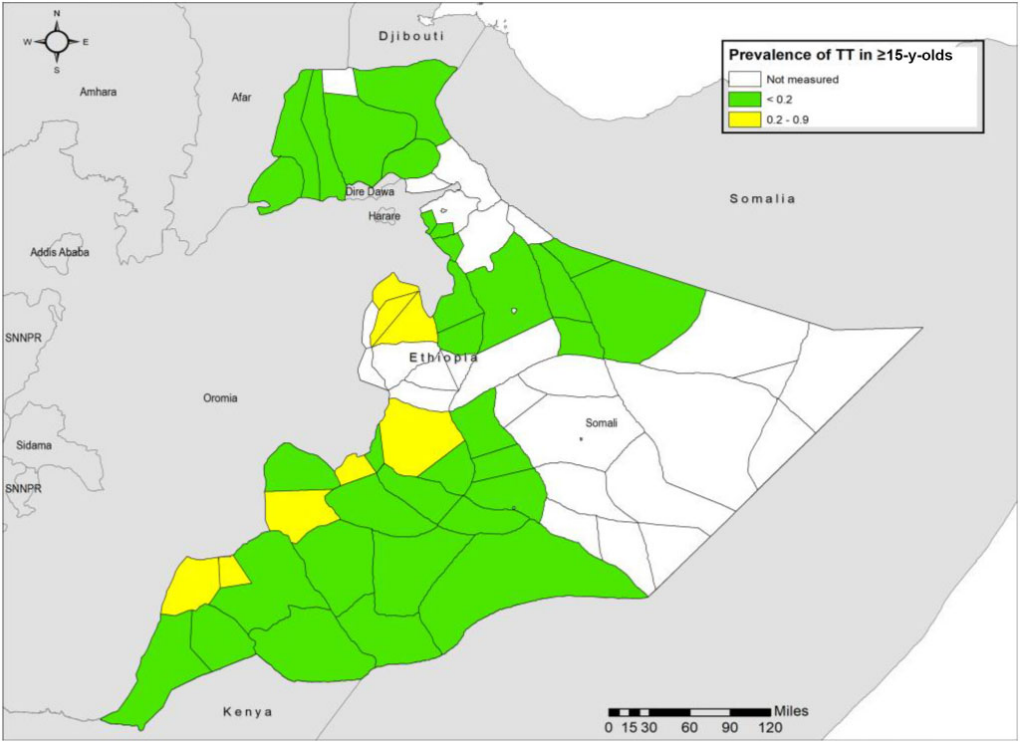
Prevalence of trachomatous trichiasis (TT) in ≥15-y-olds, in trachoma impact surveys, Somali region, Ethiopia, 2020–2022. The boundaries and names shown and the designations used on this map do not imply the expression of any opinion whatsoever on the part of the authors, or the institutions with which they are affiliated, concerning the legal status of any country, territory, city or area or of its authorities, or concerning the delimitation of its frontiers or boundaries.

### Data collection

All data were captured electronically using a purpose-built Open Data Kit-based Android smartphone application. Once informed consent was obtained for participation from the household head, Global Positioning System coordinates of the household were recorded and the recorder asked WASH-related questions of the head of the household or their proxy. Visual inspection of household WASH facilities was undertaken where relevant.

Everyone who consented was examined by a certified grader for trichiasis (upper and lower eyelid), TF and TI, using ×2.5 magnifying binocular loupes, and sunlight or a torch for illumination. To enhance the reliability of the diagnosis of TF, graders were provided with follicle size guides.^15^ Where trichiasis (upper or lower eyelid) was recorded as being present, the presence or absence of TS was also recorded, and the participant was asked if they had ever been offered management from a health worker at a primary, secondary or tertiary health unit for the eyelid found to have trichiasis; the grader also looked for evidence of a surgical scar. If the participant had received trichiasis surgery, they were asked the time since the eyelid was operated on for trichiasis and the answer was recorded.

The field team moved from house to house to collect data until the 30 selected households had been visited. Households in which ≥1 resident 1–9-y-olds were missing were revisited wherever possible before the end of the day.

People identified as having active trachoma or any likely bacterial conjunctivitis were provided with 1% tetracycline eye ointment. The survey team arranged for existing primary eye centers to receive and treat patients identified as having TT and to handle other eye conditions through appropriate onward referral.

Collected data were stored on the smartphone's micro-secure digital card, until a data-enabled mobile network or Wi-Fi signal was available and data were transmitted to the Cloud-based secure central database.

### Data analysis

TF prevalence was adjusted for age in 1-y age bands, and TT prevalence was adjusted for age and gender in 5-y age bands, using data from the most recent census.^7,13^ To explore associations between TF and individual- and household-level factors, individual level TF data were analyzed using mixed effects models. Models were tested to determine the most appropriate random effects to use, comparing the presence of TF against household, cluster and woreda, both individually and in combination. WASH variables were included as fixed effects, as were the number of children in the household, age and gender. Latrine type and drinking water source answers from the WASH questionnaire were grouped into categories of improved, unimproved and open. Washing water was not included as this correlated strongly with drinking water. Univariable models with each WASH variable were tested first, followed by a multivariable model including all significant variables. Each level of each variable was tested against a reference level within the group, and all ORs were relative to this reference level. All analyses were conducted using the statistical software R.^16^

## Results

Fieldwork took place from November 2020 to January 2022. A total of 173 644 people were enumerated in the 50 surveys, from 41 298 households in a total of 1382 clusters.

### TF prevalence

Overall, 71 031 children aged 1–9 y were examined (Table 1). The age-adjusted woreda-level prevalence of TF among 1−9-y-olds ranged from 0.2% (95% CI 0.0 to 0.6%) in Dhanan woreda to 16.2% (95% CI 10.9 to 22.4%) in Shabeley woreda. Of the 50 surveys, the prevalence of TF was <5% in 41 (82%) and ≥10% in five (10%). Four woredas had a TF prevalence in the 5–9.9% range. Details are shown in Table 2.

**Table 1.** Age distribution of survey participants, trachoma impact surveys, Somali region, Ethiopia, 2020–2022

Zone	Evaluation unit	Age, y	Examined, n	Absent, n	Refused, n	Other, n	Total, n
Afder	Elkere, Raso, West Emay, Bare, Chereti, Dolobay, God-God, Goraboqaksa, Guradamole, Hargele, Karsadulabaliad, Qooxle	1–9	18 279	83	12	—	18 374
		>15	19 085	1775	60	—	20 920
Degehabur	Ararso, Aware, Daror, Degahabure, Degahmadow, Gashamo, Gunagado, Yocale	1–9	8947	109	36	—	9092
		>15	10 999	1045	132	5	12 181
Erer	Fik, Mayamulku, Qubi, Yaxoob	1–9	5319	47	11	—	5377
		>15	5331	643	28	—	6002
Liben	Doloado, Filtu, Deka suftu, Hudet, Moyale, Mubarek, Qadaadumo	1–9	11 849	46	2	—	11 897
		>15	10 723	1262	35	—	12 020
Siti	Erer, Gablalu, Hadhagala, Shinile, Afdem, Ayshia, Dambal, Mieso	1–9	10 409	64	2	—	10 475
		>15	11 644	1344	25	—	13 013
Shebele	Adadle, Berano, Celewyane, Dhanan, East Emi, Gode	1–9	7854	37	14	—	7905
		>15	7948	1173	49	—	9170
Faafam	Babile, Goljano, Gursum, Harorays, Shabeley	1–9	8374	29	—	—	8403
		>15	7823	859	12	—	8694
Total			144 584	8516	418	5	153 523

**Table 2.** Prevalence of trachomatous inflammation—follicular (TF) in children aged 1–9 y and trachomatous trichiasis (TT) in those aged ≥15 y in trachoma impact surveys, Somali region, Ethiopia, 2020–2022

Woreda (evaluation unit)	Number of 1–9-y-olds examined	Adjusted TF prevalence in 1–9-y-olds (95% CI)^*^	Number of ≥15-y-olds examined	Adjusted prevalence of TT unknown to the health system in people aged ≥15-y-olds (95% CI)^**^	Proportion of households with an improved drinking water source	Proportion of households with an improved drinking water source within a 30-min return journey of the household	Proportion of households with an improved latrine
Elkere	1398	1.38 (0.51–2.34)	1389	0.04 (0.00–0.11)	21%	21%	32%
Raso	1335	0.54 (0.19–1.03)	1477	0.05 (0.01–0.13)	36%	26%	19%
West Emay	1313	1.41 (0.71–2.28)	1253	0 (–)	8%	21%	8%
Ararso	1019	2.43 (0.98–3.67)	1334	0.12 (0.01–0.28)	74%	40%	41%
Aware	1095	0.89 (0.28–1.71)	1379	0.05 (0.00–0.11)	58%	26%	20%
Daror	997	0.60 (0.17–1.02)	1447	0 (–)	59%	25%	32%
Degahabure	1173	0.60 (0.12–1.28)	1340	0.12 (0.03–0.25)	34%	23%	29%
Degahmadow	1243	1.77 (0.68–3.32)	1347	0.17 (0.05–0.32)	46%	22%	21%
Gashamo	1160	1.13 (0.61–1.79)	1403	0.06 (0.00–0.18)	38%	34%	34%
Gunagado	1171	0.68 (0.16–1.41)	1420	0.02 (0.00–0.05)	45%	27%	32%
Yocale	1089	0.83 (0.27–1.64)	1329	0.06 (0.00–0.18)	50%	18%	17%
Fik	1332	1.12 (0.56–1.80)	1477	0.29 (0.06–0.68)	41%	31%	29%
Mayamulku	1307	2.06 (1.09–3.30)	1271	0.39 (0.06–0.90)	27%	11%	4%
Qubi	1347	4.80 (3.44–6.15)	1262	0.21 (0.08–0.36)	16%	16%	14%
Yaxoob	1333	1.06 (0.59–1.66)	1321	0.08 (0.01–0.17)	12%	12%	9%
Doloado	1391	1.15 (0.54–1.86)	1430	0.03 (0.00–0.09)	40%	35%	46%
Filtu	1375	3.83 (2.11–6.10)	1417	0.05 (0.00–0.08)	29%	13%	28%
Adadle	1361	1.13 (0.46–1.90)	1399	0.01 (0.00–0.03)	17%	20%	23%
Berano	1241	1.56 (0.80–2.27)	1181	0 (–)	15%	26%	14%
Celewyane	1220	0.91 (0.33–1.71)	1348	0.02 (0.00–0.05)	31%	15%	7%
Dhanan	1347	0.25 (0.03–0.56)	1414	0.00 (0.00–0.00)	24%	23%	36%
East Emi	1355	1.37 (0.50–2.44)	1242	0.20 (0.00–0.56)	4%	17%	10%
Gode	1330	1.34 (0.57–2.46)	1364	0 (–)	13%	17%	17%
Afdem	1031	1.08 (0.07–2.57)	1193	0.01 (0.00–0.03)	79%	66%	47%
Ayshia	1150	0.48 (0.00–1.43)	1353	0.00 (0.00–0.00)	79%	82%	61%
Dambal	1098	0.59 (0.09–1.31)	1447	0.06 (0.00–0.14)	72%	49%	35%
Mieso	1341	1.25 (0.49–2.28)	1276	0.03 (0.00–0.07)	77%	57%	30%
Bare	1526	1.14 (0.43–2.00)	1670	0 (–)	28%	17%	19%
Chereti	1728	1.76 (0.63–2.83)	1664	0.01 (0.00–0.04)	24%	13%	21%
Dolobay	1537	1.42 (0.58–2.29)	1652	0.01 (0.00–0.03)	12%	14%	21%
God-god	1555	2.62 (0.71–5.85)	1659	0.15 (0.00–0.37)	28%	29%	12%
Goraboqaksa	1576	1.63 (0.79–2.73)	1669	0.23 (0.00–0.64)	45%	12%	35%
Guradamole	1604	3.79 (1.91–6.19)	1707	0.16 (0.02–0.34)	22%	11%	16%
Hargele	1493	1.25 (0.59–2.14)	1644	0.13 (0.03–0.29)	26%	5%	24%
Karsadulabaliad	1718	8.13 (5.26–11.23)	1584	0.27 (0.08–0.51)	17%	10%	18%
Qooxle	1496	1.38 (0.57–2.52)	1717	0.04 (0.00–0.11)	35%	25%	23%
Babile	1698	10.80 (7.55–14.53)	1588	0.11 (0.02–0.27)	51%	24%	19%
Goljano	1674	15.60 (11.46–20.56)	1533	0.07 (0.01–0.16)	67%	29%	13%
Gursum	1731	10.48 (7.23–14.64)	1544	0.12 (0.03–0.22)	60%	47%	34%
Harorays	1656	15.61 (10.87–20.04)	1627	0.12 (0.03–0.25)	64%	21%	1%
Shabeley	1615	16.23 (10.86–22.43)	1531	0.09 (0.00–0.21)	70%	20%	11%
Deka suftu	1729	5.20 (3.11–7.09)	1618	0.25 (0.06–0.49)	39%	27%	30%
Hudet	1864	5.41 (3.65–7.87)	1535	0.30 (0.02–0.61)	34%	17%	30%
Moyale	1794	4.77 (2.39–7.99)	1595	0.17 (0.04–0.38)	53%	34%	47%
Mubarek	1867	5.72 (3.61–8.61)	1550	0.09 (0.01–0.21)	24%	18%	23%
Qadaadumo	1829	2.36 (0.99–4.46)	1578	0.17 (0.04–0.35)	47%	10%	21%
Erer	1462	2.37 (1.21–3.68)	1729	0.03 (0.00–0.07)	56%	53%	41%
Gablalu	1406	1.95 (0.88–2.83)	1338	0.00 (0.00–0.01)	38%	18%	2%
Hadhagala	1504	1.84 (0.56–3.66)	1604	0.03 (0.00–0.10)	76%	72%	49%
Shinile	1417	1.52 (0.68–2.63)	1704	0.05 (0.00–0.14)	72%	53%	51%

* Adjusted in 1-y age bands according to the 2007 census.^13^

** Adjusted in 5-y age and gender bands according to the 2007 census.

### TT prevalence

A total of 73 553 adults aged ≥15 y were examined (Table 1). A total of 318 TT cases, of which 234 were unknown to the health system, were identified in this group. The age- and gender-adjusted prevalence of TT unknown to the health system in ≥15-y-olds ranged up to 0.39% (95% CI 0.06 to 0.90%). This metric was <0.2% in 42 of 50 surveyed woredas (Table 2).

### WASH access

A total of 41 298 households were visited; 18% (n=7588) had access to improved drinking water within a 30-min journey. Only 25% (n=10 362) of households had an improved latrine, and 17% (n=6895) of households had a latrine with a handwashing station (Table 2).

### Associated factors

Household alone was identified as being the most appropriate random effect for these data. Univariable models showed that age, gender and the number of children per household were significantly (p<0.001) associated with TF prevalence, with ORs suggesting that children aged 1–3 y had 51-fold increased odds of having TF compared with people aged ≥16 y; females were slightly less likely than males to have TF, and having ≥2 children in the household doubled the odds of TF. The multivariable model suggested that the number of children in the household was not significant when age and gender were also considered (Table 3).

**Table 3.** Association between trachomatous inflammation—follicular (TF) prevalence in 1–9-y-olds and individual- and household-level variables in trachoma impact surveys, Somali region, Ethiopia, 2020–2022

Variable		Univariable OR (95% CI)	p	Multivariate OR (95% CI)	p
Age (y)	1–3	51.3 (41.5–63.2)	<0.001	54.6 (44.1–67.9)	<0.001
	4–6	37.9 (30.7–46.7)	<0.001	37.8 (30.5–47.0)	<0.001
	7–9	15.2 (12.1–19.0)	<0.001	17.3 (13.8–21.9)	<0.001
	10–15	2.8 (2.1–3.7)	<0.001	3.4 (2.6–4.5)	<0.001
	>16	Reference	-	-	-
Gender	Female	0.7 (0.6–0.8)	<0.001	0.7 (0.7–0.8)	<0.001
	Male	Reference	-	-	-
Handwash station	Yes	1.1 (0.6–2.0)	0.635	-	-
	No	Reference	-	-	-
Number of children aged 1–9 y per household	0–1	Reference	-	-	-
	2–3	2.2 (1.2–3.9)	0.008	0.6 (0.3–1.1)	0.09
	4–5	2.2 (1.1–4.3)	0.03	0.5 (0.2–1.1)	0.08
	**≥**6	1.9 (0.2–16.7)	0.6	0.2 (0.0–2.5)	0.19
Drinking water source	Improved	Reference	-	-	-
	Unimproved	0.9 (0.5–1.5)	0.6	-	-
	Surface	1.1 (0.6–1.7)	0.8	-	-
Distance to nearest drinking water source (min)	>30	Reference	-	-	-
	<30	0.9 (0.6–1.4)	0.7	-	-
Improved latrine	Improved	Reference	-	-	-
	Unimproved	1.4 (0.5–3.7)	0.5	-	-
	Open	1.33 (0.8–2.3)	0.3	-	

## Discussion

Encouraging progress has been made to reduce the prevalence of TF and TT in the Somali region of Ethiopia. In this series of impact surveys, TF prevalence was <5% in 41 of 50 surveyed woredas and the prevalence of TT unknown to the health system was below the WHO elimination threshold level of 0.2% in 42 of 50 woredas. However, WASH coverage was poor, with only 18% of households having access to improved drinking water within a 30-min round trip and 25% of households having an improved latrine.

As per WHO recommendations, three more rounds of annual antibiotic MDA are required in the five woredas that have a TF prevalence ≥10% in children aged 1–9 y. One more round of MDA is indicated in the four woredas found to have a TF prevalence of 5–9.9%. In addition, efforts to implement the F and E components of the SAFE strategy (or, more generally, improve access to WASH) are needed across all of the area surveyed, not just for trachoma elimination purposes.^17,18^ In the 41 woredas that have met the WHO-recommended TF elimination target, further rounds of antibiotic MDA are not needed, but trachoma surveillance surveys should be conducted at least 2 y after the impact surveys took place, in order to check for recrudescence.^19^

Reductions in TF prevalence compared with baseline^6^ following the previous rounds of MDA were observed in a large proportion of the woredas surveyed here. This is pleasing: although reducing TF prevalence (and thereby reducing the risk of future trachomatous blindness by minimizing the deposition of conjunctival scar) is the desired outcome, the impact of MDA in Ethiopia has been more variable than elsewhere in Africa.^20–22^ Reasons for the relative success in Somali region could include good MDA coverage, low trachoma prevalence at baseline,^6^ or other factors. Further research would be required to understand the likely explanation with any confidence. We also acknowledge that five woredas require at least 3 more years of interventions against active trachoma, and four woredas require at least 1 additional year; the battle against trachoma here is not yet won.

This study had a number of strengths. We employed a standardized methodology that conforms with WHO recommendations^9^ and incorporates quality assurance processes at every step of the survey process.^7,8^ Also, the required sample size for TF in children aged 1–9 y^9^ was met for all woredas, increasing the precision around the TF prevalence estimates. However, there are also some limitations. The minimum sample size of 2818 for TT in adults aged ≥15 y was not met in any woredas, although 30 clusters were surveyed for the 23 woredas surveyed in 2021–2022, which statistical modeling has indicated provides sufficient precision around the TT prevalence estimate.^14^ We also encountered some difficulties with population movement and insecurity in the survey area. These issues were mitigated through close communication with the field teams to locate displaced populations, and in instances where villages no longer existed, replacing them with newly selected clusters. Insecurity did lead to some delays in completing fieldwork as cluster replacement can affect the geographical representativeness of the sample and introduce bias. Field team security is the primary concern and, as with the GTMP, surveys are not undertaken if field teams are put at a security risk.^8^

In conclusion, there has been good progress in reduction of TF and TT prevalence in the woredas surveyed here. However, trachoma remains a public health problem in Somali region. Continuing implementation of the SAFE strategy, maintaining TF prevalence reductions through behavioral activity intervention, ensuring the existence of a system to identify and manage incident cases of TT^23^ and conducting surveillance surveys after a 2-y interval in woredas shown to have TF prevalence below the elimination threshold,^19^ will all be critical for program success and long-term sustainability.

## Data Availability

The data used in this paper are owned by the Ethiopia Ministry of Health. Data are available through reasonable request to the corresponding author.
